# Recent Advances in Selective Laser Melting of Cobalt-Free Eutectic High-Entropy Alloys: Design, Microstructure, and Performance Control

**DOI:** 10.3390/mi17050536

**Published:** 2026-04-28

**Authors:** Xiaojun Tan, Xuyun Peng, Wei Tan, Jian Huang, Chaojun Ding, Yushan Yang, Jieshun Yang, Haitao Chen, Liang Guo, Qingmao Zhang

**Affiliations:** 1 Guangdong Engineering Center for Intelligent Sensing and Flexible Manufacturing, Sino-German Intelligent Manufacturing School, Shenzhen City Polytechnic, Shenzhen 518116, China; tanxiaojun920@163.com (X.T.); 18620373551@163.com (W.T.); hejson@163.com (J.H.); 18617076210@163.com (C.D.); real210@163.com (Y.Y.); yangjieshun@126.com (J.Y.); a202415814644653@163.com (H.C.); 2 Guangdong Provincial Key Laboratory of Nanophotonic Functional Materials and Devices, School of Information and Optoelectronic Science and Engineering, South China Normal University, Guangzhou 510006, China; guoliangchn@163.com (L.G.); zhangqm@scnu.edu.cn (Q.Z.)

**Keywords:** high-entropy alloy, cobalt-free, selective laser melting, eutectic strengthening, post-processing treatment

## Abstract

With the strategic shift toward reducing reliance on critical raw materials, Cobalt-free eutectic high-entropy alloys (EHEAs) have emerged as a pivotal frontier for high-performance structural applications. This review systematically elucidates the synergistic relationship between Co-free alloy design and the non-equilibrium solidification mechanisms of Selective Laser Melting (SLM). The ultra-high cooling rates (10^5^–10^8^ K/s) inherent in SLM are shown to refine eutectic lamellae to the sub-micron scale (typically <300 nm), effectively suppressing the macro-segregation common in conventional casting. We evaluate the design principles of Al-Cr-Fe-Ni and related systems, noting that SLM-processed Co-free EHEAs frequently achieve yield strengths exceeding 1000 MPa and ultimate tensile strengths (UTSs) surpassing 1300 MPa, while maintaining tensile elongations above 10%—a significant improvement over the coarse-grained structures produced by traditional methods. Furthermore, the study identifies critical processing windows, such as laser energy densities (60–120 J/mm^3^), required to mitigate micro-cracking and achieve near-full density (>99.5%). By synthesizing recent experimental breakthroughs and AI-driven modeling, this review provides a quantitative roadmap for the precision manufacturing of cost-effective, high-performance EHEAs, bridging the gap between theoretical alloy design and industrial additive manufacturing.

## 1. Introduction

### 1.1. Conceptual Evolution and Definition of HEAs

High-entropy alloys (HEAs), pioneered by Yeh et al. [1] and Cantor et al. [2], represent a transformative paradigm in metallurgy, shifting the focus from traditional dilute solutions to concentrated multi-principal element systems. Conventionally, HEAs are identified by two synergistic criteria: (i) the compositional definition, requiring at least five principal elements with atomic concentrations ranging from 5% to 35% [3,4]; and (ii) the thermodynamic definition, characterized by a molar configurational entropy (ΔS_conf_) exceeding 1.5 R in a random solid solution state [5]. This high-entropy effect effectively minimizes the Gibbs free energy of the system (ΔGmix = ΔHmix − TΔSmix), thereby promoting the formation of simple solid solution phases (e.g., FCC, BCC, or HCP) while kinetically suppressing the precipitation of complex, brittle intermetallic compounds [6].

However, as summarized in Table 1, the compositions do not strictly satisfy the conventional HEA definition, as their configurational entropy is <1.5 R and the number of principal elements is fewer than five. To accommodate evolving application-oriented designs—particularly the emerging class of Eutectic High-Entropy Alloys (EHEAs)—this work adopts a more inclusive and practical definition: alloys containing at least four principal elements, each with a concentration exceeding 10 at.%. This expanded scope is instrumental for EHEAs, which bridge the gap between HEA theory and dual-phase microstructural engineering. In these systems, the high-entropy effect stabilizes the constituent solid solution phases, while the eutectic composition ensures the superior castability and printability necessitated by Selective Laser Melting (SLM).

In the context of SLM, the transition to non-equimolar compositions is a deliberate metallurgical strategy rather than a mere deviation. As demonstrated by Su et al. [7], fine-tuning the Al content in the Al-Cr-Cu-Fe-Ni system away from strict equimolarity can effectively compensate for the selective laser-induced evaporation of volatile elements and precisely regulate the phase constitution. Such off-eutectic optimization, when coupled with the extreme cooling rates of SLM, facilitates the formation of refined hierarchical structures and metastable phases that are unattainable via conventional near-equilibrium casting.

By tailoring elemental combinations and compositional ratios, HEAs exhibit excellent high-temperature thermal stability, while simultaneously maintaining high strength, ductility, and fracture toughness at low temperatures [8]. In addition, HEAs may demonstrate remarkable properties such as super-para-magnetism [9], superconductivity [10], and outstanding irradiation resistance [11].

**Table 1:** Some HEAs fabricated by SLM.

HEA	Configurational Entropy	Phase	Reference
FeCoCrNi	1.39 R	FCC	[12]
Ni6Cr4WFe9Ti	1.33 R	FCC	[13]
CrCuFeNi2	1.33 R	FCC	[7]
Al0.9Cr0.9Fe2.1Ni2.1	1.30 R	FCC + BCC	[14]
WMoTaTi	1.38 R	BCC + HCP	[15]
Fe49.5Mn30Co10Cr10C0.5	1.20 R	FCC + HCP	[16]
NbMoTaW	1.39 R	BCC	[17]
CrFeNiMn	1.39 R	FCC	[3]
FeCoCrNi	1.39 R	FCC	[18]
FeCoNiCr0.5	1.35 R	FCC	[19]
Fe50Mn30Co10Cr10	1.17 R	FCC	[20]

### 1.2. Strengthening Potential and Performance Benchmarks of HEAs

**Figure 1:**
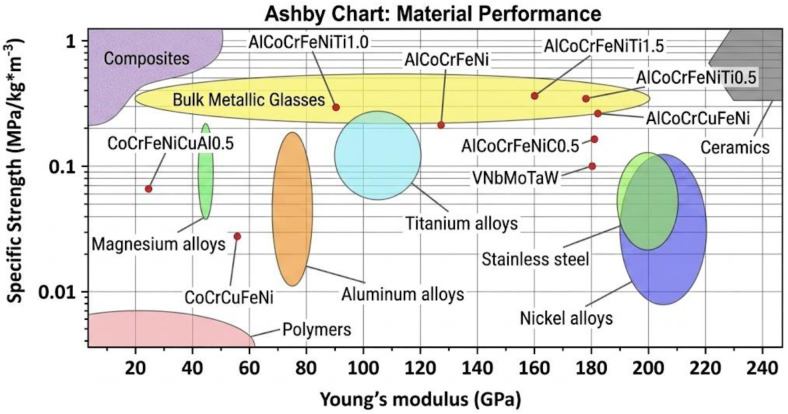
Specific strength and Young’s modulus of traditional alloys and HEAs [21].

High specific strength is a defining attribute of HEAs, providing the fundamental basis for their application in extreme environments such as high-temperature mold systems [6]. As illustrated in Figure 1, many HEAs surpass conventional titanium and nickel-based alloys in specific strength due to the “severe lattice distortion effect” and the stabilization of high-strength secondary phases [21]. However, achieving this potential requires precise navigation of processing routes and microstructural tuning.

Recent advancements in conventional metallurgy have demonstrated the efficacy of complex phase regulation. For instance, Wang et al. [22] utilized CALPHAD-guided design to develop TiZrHf-based entropic alloys with an α + β dual-phase microstructure. This approach leverages the transformation-induced plasticity (TRIP) effect to achieve an exceptional balance between tensile strength, corrosion resistance, and high-temperature oxidation stability. Furthermore, post-processing techniques such as cryogenic aging have emerged as powerful tools for microstructural refinement. Liu et al. [23] demonstrated that cryogenic treatment can effectively regulate precipitates and dislocation density, enhancing the tensile strength of Al-based alloys to 512.5 MPa—outperforming several additive manufacturing benchmarks.

While these traditional thermo-mechanical treatments provide significant enhancement, they often struggle to achieve the extreme grain refinement and chemical homogeneity required for Cobalt-free EHEAs. The inherent challenge in Co-free systems—such as AlCrFeNi—lies in their sensitivity to cooling rates and elemental segregation. This underscores the necessity of transitioning from near-equilibrium casting to SLM. By harnessing the ultra-fast solidification kinetics of SLM, it is possible to bypass the limitations of conventional processing, directly capturing the sub-micron hierarchical structures and supersaturated solid solutions that drive the next generation of high-strength, sustainable HEAs.

### 1.3. Primary Strengthening Strategies for SLM-Fabricated HEAs

**Figure 2:**
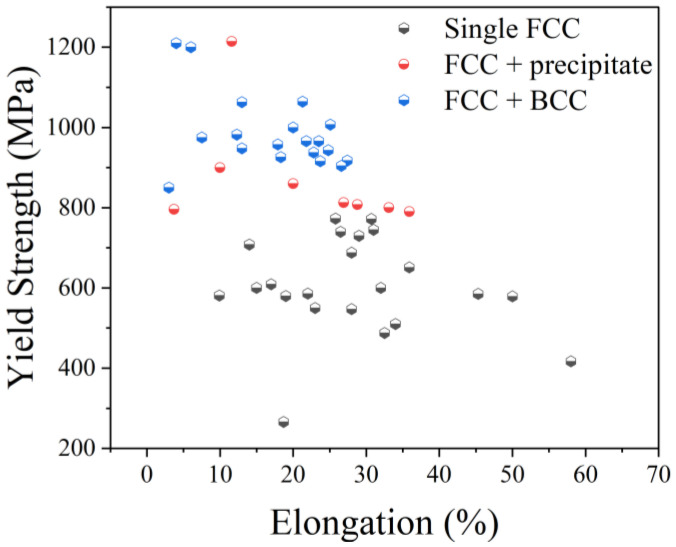
Relationship between phase composition, tensile yield strength, and elongation of SLM-processed HEAs [8,12,19,24,25,26,27,28,29,30,31,32,33,34,35,36,37,38,39,40,41,42,43,44,45,46,47,48,49,50,51,52].

Figure 2 and Table 2 summarize the microstructures and tensile properties of representative SLM-fabricated HEAs reported in the recent literature. Historically, single-phase face-centered cubic (FCC) HEAs have been favored for their excellent ductility (typically δ > 10%); however, their relatively low yield strength (YS < 800 MPa) remains a significant barrier to structural applications. Enhancing the strength of these single-phase systems without sacrificing toughness has thus emerged as a critical challenge in additive manufacturing.

Based on current research, three primary strategies are employed to strengthen SLM-fabricated HEAs: (1) Particle strengthening, via the introduction of ceramic or oxide reinforcements; (2) Nanoscale precipitation strengthening, utilizing the intrinsic heat treatment (IHT) effect of SLM to trigger fine phase separation; and (3) Eutectic structural strengthening, which leverages the dual-phase (FCC + BCC/B2) hierarchy to create high-density phase boundaries.

In multi-principal element systems such as the Al-Cr-Fe-Ni-based series, the efficacy of strengthening strategies is uniquely dependent on compositional precision and processing control. As synthesized in Table 2, a sensitive correlation exists between the aluminum (Al) molar ratio and the fraction of the secondary hard B2 phase. When the Al ratio exceeds a critical threshold (typically 0.7), the yield strength of the alloy increases significantly—by approximately 40%—driven by the microstructural transition from a single-phase FCC matrix to a refined eutectic hierarchy. This transition, however, necessitates a rigorous trade-off with ductility, as the intrinsically brittle nature of the B2 phase can initiate premature cracking if the microstructural scale is not properly refined through rapid solidification.

Furthermore, the mechanical performance of SLM-fabricated HEAs is highly susceptible to variations in laser energy density. A comparative analysis of the Al-Co-Cr-Fe-Ni system in Table 2 (Ref. [26]) reveals a significant gap in Ultimate Tensile Strength (UTS) primarily due to differences in relative density (>99% vs. 96%). This underscores the critical role of melt pool dynamics; specifically, adjusting the elemental ratios (such as removing or substituting specific components) modifies the melt’s surface tension and viscosity, which can significantly narrow the “printability window.” Achieving high strength in these advanced alloys, therefore, requires a synergistic approach that balances thermodynamic phase stability with precise laser-matter interaction to ensure both compositional homogeneity and structural integrity.

#### 1.3.1. Particle Strengthening in SLM-Fabricated HEAs

**Figure 3:**
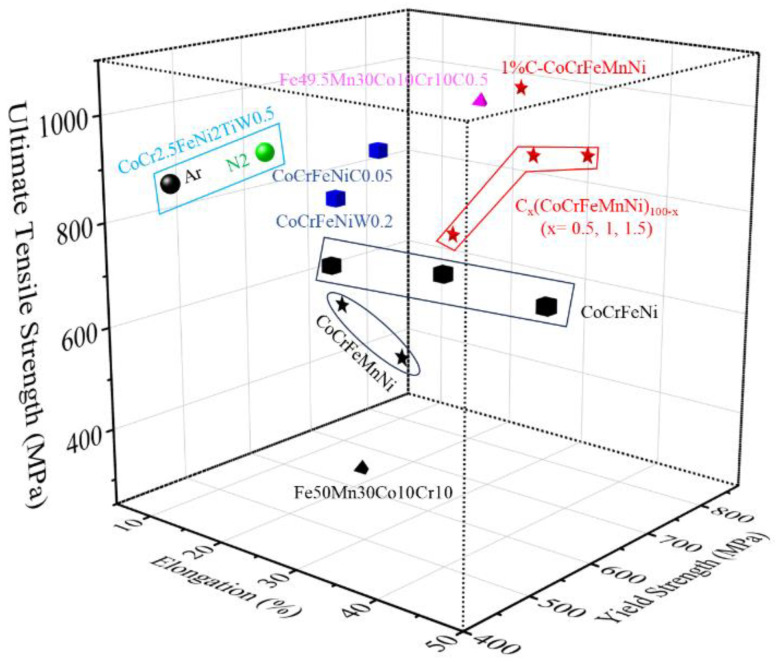
Examples of SLM-processed HEAs reinforced by carbon/nitride and tungsten particles (black ones are as-SLMed HEAs without carbon/nitride or tungsten particles, other colors and shapes are as-SLMed HEAs with carbon/nitride or tungsten particles) [18,20,26,29,30,31,32,53,54,55,56,57,58].

Figure 3 presents cases of particle-strengthened SLM-fabricated high-entropy alloys (HEAs). The addition of particles significantly enhances both the yield strength (YS) and ultimate tensile strength (UTS). Based on the type of particles used, these can be categorized into nitride, carbide, and tungsten particle strengthening.

**Figure 4:**
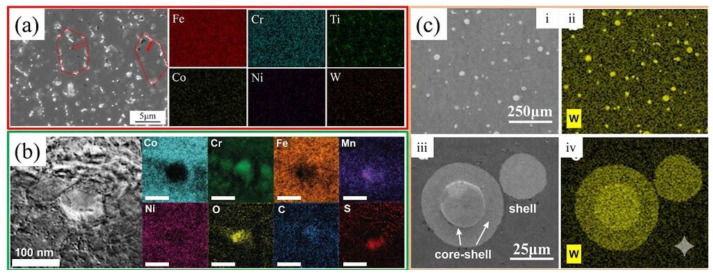
Microstructure of SLM-processed HEAs reinforced by carbon/nitride and tungsten particles: (**a**) CoCr2.5FeNi2TiW0.5 (the red area is the shape of the crystal; the solid red arrow represents the precipitate gathered at the grain boundary and the red hollow arrow represents the precipitate within the grain) [32]; (**b**) 1%C-CoCrFeMnNi [59]; (**c**) CoCrFeNiW0.2 ((i,iii) SEM secondary electron image of AF and HT1 samples respectively. Micrographs in (i,iii) were examined using EDX elemental mapping of W, Co, Cr, Fe and Ni in (ii,iv)) [53].

Nitride Particle Strengthening: Gu et al. [32] found that CoCr2.5FeNi2TiW0.5 prepared in a nitrogen atmosphere formed TiN nanoparticles (Figure 4a), resulting in higher strength compared to the same alloy prepared in an argon atmosphere.

Carbide Particle Strengthening: In the C-CoCrFeMnNi alloy fabricated by Park et al. [59], carbide nanoparticles were distributed along the edges of the cellular structures (Figure 4b), enhancing both yield and tensile strength. Zhu et al. [16] prepared an Fe49.5Mn30Co10Cr10C0.5 alloy via SLM, achieving a yield strength of 710 MPa—significantly higher than the 630 MPa reported for carbon-free Fe50Mn30Co10Cr10 [20].

Tungsten Particle Strengthening: Ng et al. [53] mixed tungsten powder with CoCrFeNi powder to fabricate alloys containing unmelted W particles (Figure 4c), reaching a YS of 610 MPa and a UTS of 814 MPa.

While particle strengthening effectively improves alloy strength, the poor metallurgical compatibility between the matrix and the particles may affect the long-term stability of the composite. For instance, mismatched coefficients of thermal expansion can lead to cracking or deformation under various operating environments. Furthermore, these composites are susceptible to temperature and humidity fluctuations over prolonged use, potentially leading to embrittlement or intergranular corrosion [58].

#### 1.3.2. Precipitation Strengthening in SLM-Fabricated HEAs

**Figure 5:**
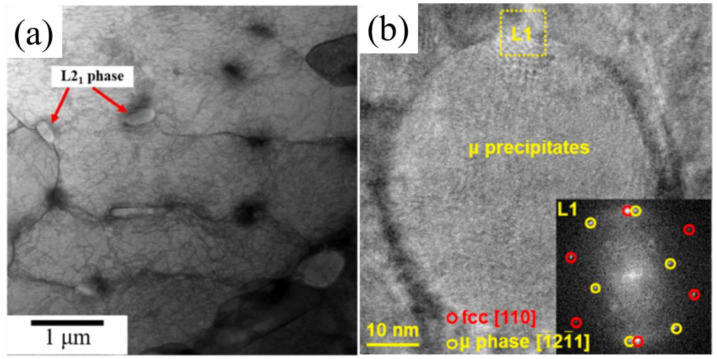
Precipitation of SLM-processed HEAs: (**a**) Al0.2Co1.5CrFeNi1.5Ti0.3 [44]; (**b**) (CoCrNi)95Mo5 [46].

As shown in Figure 2, the yield strength of HEAs containing precipitates is generally higher than that of single-phase FCC alloys. Regarding precipitation strengthening, researchers have promoted the formation of secondary phases by adding specific elements. For example, Lin [43] and Ikeda [44] prepared alloys such as Al0.2Co1.5CrFeNi1.5Ti0.3 and (CoCrNi)95Mo5, where precipitates—including the L2_1_ phase (Figure 5a) and the μ phase (Figure 5b)—significantly enhanced the strength.

However, the formation and distribution of precipitates are highly sensitive to the SLM processing environment. Fluctuating processing conditions can affect the composition and uniformity of the precipitates, thereby exerting an adverse impact on the mechanical properties of the alloy [60].

#### 1.3.3. Eutectic Structure Strengthening in SLM-Fabricated HEAs

The yield strength of eutectic high-entropy alloys (EHEAs) is generally above 800 MPa, with some even exceeding 1200 MPa, far outperforming single-phase FCC HEAs (Figure 2). Ren et al. [25] fabricated a dual-phase nanolamellar AlCoCrFeNi2.1 HEA via SLM, which achieved a YS of 1063 MPa, a UTS of 1386 MPa, and an elongation of 13%. Similarly, Guo [26] and He [27] prepared AlCoCrFeNi2.1 alloys with yield strengths exceeding 950 MPa and UTS exceeding 1250 MPa.

In summary, strengthening SLM-fabricated cobalt-free HEAs through eutectic structures can significantly improve both strength and ductility. This approach holds broad application prospects, particularly with immense potential in fields such as high-temperature dies and molds.

Unlike existing reviews that broadly cover the general additive manufacturing of HEAs, this work establishes a unifying ‘Composition-Process-Structure-Property’ (CPSP) framework specifically for Cobalt-free EHEAs. The novelty of this review lies in:

(1) elucidating the Co-free design principles that bypass strategic resource dependencies;
(2) synthesizing the non-equilibrium solidification physics of SLM (cooling rates of 10^5^–10^8^ K/s) as a tool for sub-micron lamellar refinement;
(3) identifying the critical knowledge gap between theoretical CALPHAD predictions and the actual hierarchical heterostructures formed during rapid melting.

By mapping these interdependencies, this review provides a predictive roadmap for achieving superior strength-ductility balance in cost-effective EHEAs.

## 2. Challenges in the Synthesis and Powder Preparation of Co-Free EHEAs

The transition from Co-containing to Co-free eutectic high-entropy alloys (EHEAs) is not merely a compositional substitution but a fundamental shift in the metallurgical processing window. Unlike traditional HEAs, the absence of Cobalt significantly alters the thermophysical properties of the melt, posing unique challenges during both ingot smelting and subsequent powder atomization for SLM [13].

### 2.1. Impact of Cobalt-Free Composition on Melt Behavior

Cobalt plays a crucial role in stabilizing the liquid phase and modulating the viscosity of multi-principal element melts. In Co-free systems, such as the Al-Cr-Fe-Ni series, the removal of Co typically leads to [3]:

Increased Melt Viscosity: The loss of Co disrupts the atomic packing density in the liquid state, often increasing viscosity. This hampers the homogenization of high-melting-point elements (e.g., Cr and Fe) during vacuum arc melting (VAM), potentially leading to micro-segregation in the master ingot.

Shifted Eutectic Points: The Co-free landscape narrows the “eutectic valley.” Minor deviations in processing temperatures or cooling rates can shift the solidification path from a coupled eutectic growth to a primary dendritic growth, which is detrimental to the uniform sub-micron structure required for SLM.

### 2.2. Elemental Evaporation and Stoichiometric Control

During vacuum induction melting (VIM) or arc melting of Co-free EHEAs, the vapor pressure mismatch between constituents becomes more pronounced.

Selective Evaporation of Volatile Elements: Elements like Aluminum (Al) exhibit significantly higher vapor pressures compared to Fe or Ni. Without the buffering effect of Cobalt on the thermodynamic activity of Al, the “compositional drift” becomes harder to predict.

Implications for SLM: Since the eutectic balance in Co-free systems is highly sensitive to the Al/Ni ratio, even a 1–2 at. % loss during smelting can result in the formation of brittle pro-eutectic B2 phases, increasing the cracking susceptibility during the subsequent laser melting process [14].

### 2.3. Powder Atomization Requirements for Co-Free EHEAs

The quality of SLM parts is highly dependent on the precursor powder. For Co-free EHEAs, the gas atomization process must be specifically tuned:

Fluidity and Surface Tension: The altered surface tension of Co-free melts affects the “break-up” mechanism during atomization. Higher gas-to-melt ratios are often required to achieve the desired spherical morphology and a narrow particle size distribution (typically 15–53 µm).

Satellite Formation: Due to the modified cooling kinetics of Co-free droplets, there is a higher tendency for “satellite” attachment if the atomization tower parameters are not optimized for the specific heat capacity of the Al-Cr-Fe-Ni system [15].

## 3. Current Research Status of SLM-Fabricated Cobalt-Free High-Entropy Alloys

In the existing literature, most HEAs fabricated by SLM contain cobalt. Owing to its excellent high-temperature performance and strengthening effects, cobalt has become a key element in many HEA systems. However, cobalt resources in China are relatively scarce and largely dependent on imports, with limited and unstable supply channels. Consequently, the development of cobalt-free HEAs has emerged as an important research topic in materials science.

**Figure 6:**
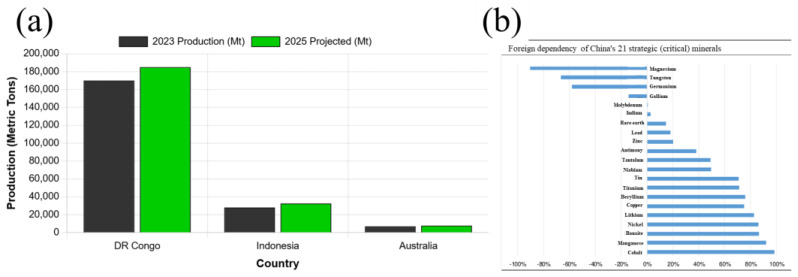
(**a**) Cobalt Production by Country (2023 vs. 2025) (StatRanker.org); (**b**) China’s dependence on foreign countries for 21 strategic (critical) minerals [61].

According to data from the U.S. Geological Survey (USGS), as of 2022, China’s proven recoverable cobalt reserves amounted to only 140,000 tons, accounting for 1.69% of global reserves. These resources mainly occur as associated minerals in iron, copper, and nickel ores, making separation technically challenging. Cobalt resources in China are primarily concentrated in Gansu Province. In 2022, China’s cobalt production was approximately 2200 tons, representing only 1.16% of global production (Figure 6a). Data from Minmetals Securities indicate that China’s external dependence on cobalt ore reaches as high as 98.41%, ranking first among strategic mineral dependencies (Figure 6b). Such a high degree of reliance, combined with limited import sources, exposes China to risks of cobalt shortages or supply disruptions. Moreover, cobalt is expensive, particularly in applications such as ternary lithium batteries in the new energy industry, where cobalt contributes significantly to material costs. Therefore, the development of cobalt-free HEAs holds substantial commercial potential. Alternative elements commonly used in HEAs, such as Ni, Cr, and Fe, are relatively more economical.

**Figure 7:**
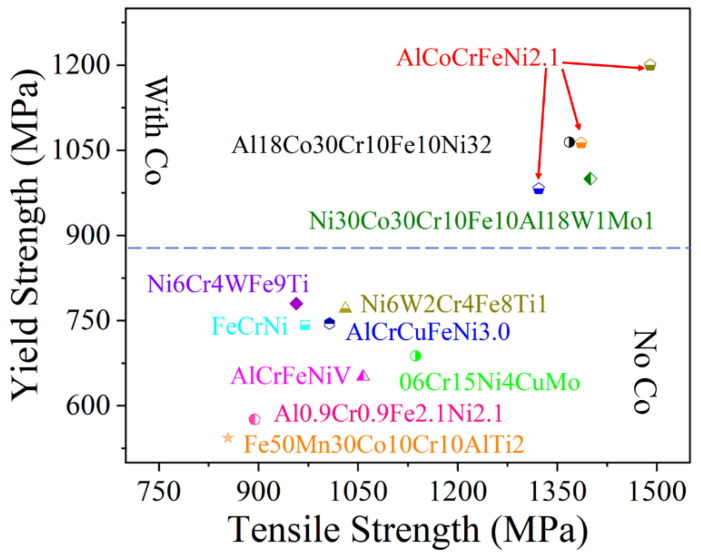
Comparison of the strength of Co-free HEAs and Co-based HEAs [8,13,14,25,26,27,28,62,63,64,65]. (Three red arrows point to AlCoCrFeNi2.1 from different references).

Although several studies have reported SLM-fabricated cobalt-free HEAs or medium-entropy alloys [7,21,24], their strength remains significantly lower than that of cobalt-containing HEAs, thereby limiting their application in high-strength and high-temperature environments (as shown in Figure 7). To promote the broader application of cobalt-free HEAs, it is therefore imperative to systematically investigate strategies for significantly enhancing their mechanical properties—particularly high-temperature strength and long-term durability—through optimized alloy composition design, tailored SLM processing parameters, and appropriate post-processing treatments.

Advancing this research direction will not only reduce dependence on strategic cobalt resources but also facilitate the expansion of cobalt-free HEAs into broader industrial applications, thereby promoting innovation and development in advanced materials technologies. In particular, optimizing the performance of cobalt-free HEAs for use in high-temperature mold systems carries considerable practical significance.

### 3.1. Research Status of Eutectic High-Entropy Alloy Powder Preparation

In the study of SLM-fabricated eutectic high-entropy alloys (EHEAs), powder properties directly dictate the quality and performance of components produced via additive manufacturing (AM) techniques such as Powder Bed Fusion (PBF) and Laser Engineered Net Shaping (LENS). Poor powder quality can lead to defects such as porosity, cracking, inclusions, and suboptimal surface roughness [66]. Currently, the powders used for SLM of EHEAs are primarily categorized into three types: gas-atomized pre-alloyed powders, mixtures of gas-atomized alloy powders and elemental powders, and uniformly mixed elemental powders.

#### 3.1.1. Gas-Atomized Pre-Alloyed EHEA Powders

**Figure 8:**
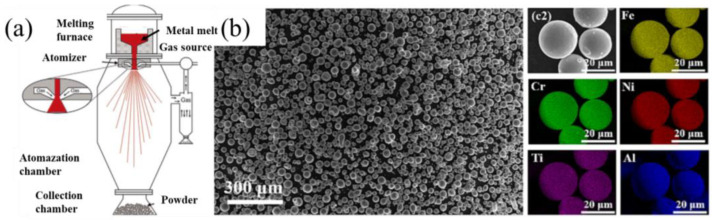
Gas atomization device and the resulting powder: (**a**) Schematic diagram of a typical experimental setup for gas atomization and its atomization stage [66]; (**b**) Morphology and element distribution of gas-atomized (FeCrNi)94Ti3Al3 HEA powders [51].

Gas atomization is the most common method for preparing powders for SLM-fabricated EHEAs. The principles of gas atomization and the resulting powder morphology are shown in Figure 8. Both gas and water atomization processes can directly produce spherical powders with excellent processability. In contrast, powders prepared by mechanical alloying often exhibit irregular shapes and require post-processing to improve sphericity [66]. At present, gas-atomized pre-alloyed powders remain the preferred choice for EHEA research.

In addition to pre-alloyed powders, Table 3 summarizes other preparation methods for HEA powders (some of which are eutectic), mainly including the “gas-atomized + elemental powder” mixing method and the “fully elemental powder” mixing method.

#### 3.1.2. Mixed Gas-Atomized and Elemental Powders

Some researchers prepare EHEA powders by adding elemental powders to gas-atomized pre-alloyed bases. For instance, Chen et al. [67] prepared CoCrFeMnNi HEAs by mixing gas-atomized CoCrFeNi pre-alloyed powder with Mn powder, achieving high-quality SLM prints. Wang et al. [68] fabricated HEA-TiAl by adding TiAl powder to gas-atomized CoCrFeMnNi powder, further enhancing performance through hot isostatic pressing (HIP) and aging, which significantly improved yield and ultimate tensile strength. Hou et al. [20] introduced Si into Fe50Mn30Co10Cr10 pre-alloyed powder to create EHEAs with varying Si content; results showed that increasing Si content markedly enhanced both strength and UTS. Vogiatzief et al. [14] successfully fabricated crack-free AlCrFeNi EHEAs by mixing AlCrFe2Ni2 pre-alloyed powder with elemental Fe and Ni, optimizing the porosity of the printed material.

#### 3.1.3. Uniformly Mixed Elemental Powders

The use of purely elemental powder mixtures has also been explored. Liu et al. [69] compared SLM and LENS in the in situ alloying of CrMoTi medium-entropy alloys, finding that SLM-fabricated samples had lower density and hardness than those produced by LENS. Furthermore, unmelted Mo powder was observed in SLM samples, indicating that high-melting-point powders struggle to melt completely during the SLM process. However, Sun et al. [33] successfully prepared AlCoCrFeNi EHEAs using this method, achieving a YS of 540 MPa, a UTS of 878 MPa, and an elongation of 18%, demonstrating the feasibility of the approach.

In summary, while gas-atomized pre-alloyed powder is the standard, mixed powder methods offer potential for modifying alloys that are otherwise difficult to process. When using elemental mixtures, attention must be paid to the melting point differentials—particularly for refractory elements. Pre-alloying remains an effective strategy to lower melting points and improve SLM processability.

### 3.2. Research Status of Composition Design for SLM-Fabricated EHEAs

**Figure 9:**
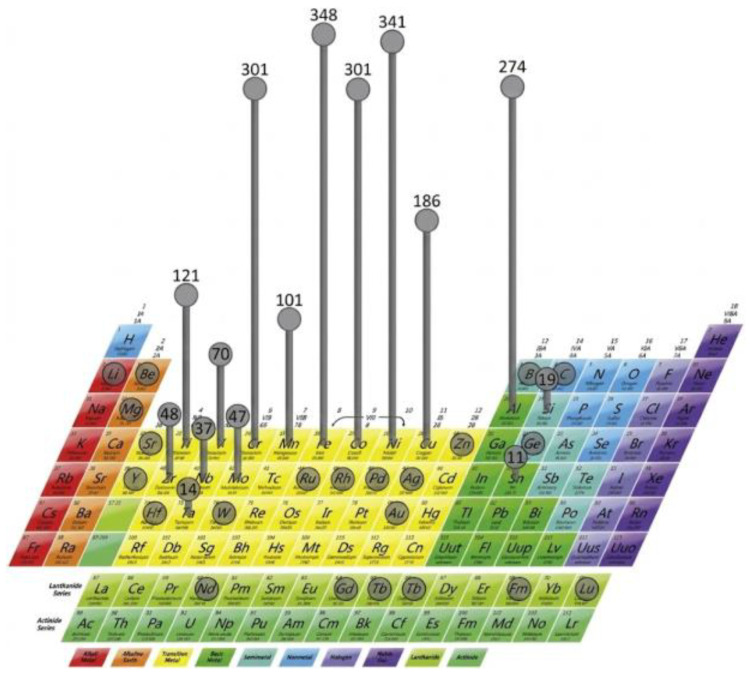
Number of uses of elements in 408 HEAs [6].

Composition design is critical to the microstructure and performance of HEAs in SLM. Miracle et al. [6] analyzed the frequency of element usage in HEAs, identifying Fe, Ni, Cr, Co, Al, Cu, and Ti as the most widely used, while high-melting-point elements like V, Mo, Zr, and Nb are often added in trace amounts (Figure 9). Consequently, current EHEA research focuses primarily on systems composed of Fe, Ni, Cr, Co, Al, Cu, and Ti. Design methodologies typically involve experimental studies and machine learning (ML).

#### 3.2.1. Experimental Research

Researchers have explored the effects of various components on the formability and properties of SLM-fabricated alloys, with a focus on adjusting Al and Ni content.

**Figure 10:**
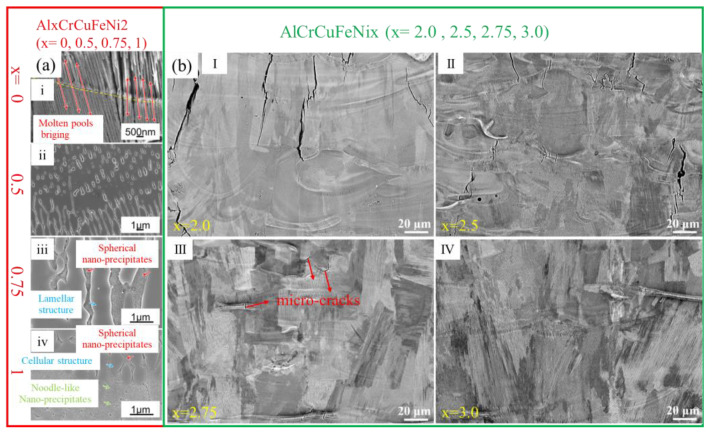
Microstructure of SLM-processed EHEAs with composition: (**a**) AlxCrCuFeNi2 (x = 0, 0.5, 0.75, 1) microstructure ((i) Al0 alloy; (ii) Al0.5 alloy; (iii) Al0.75 alloy; (iv) Al1.0 alloy) [7]; (**b**) AlCrCuFeNix (x = 2.0, 2.5, 2.75, 3.0) microstructure ((I) x = 2.0; (II) x = 2.5; (III) x = 2.75; (IV) x = 3.0) [70].

(1) Effect of Al Content: Su et al. [7] studied AlxCrCuFeNi2 HEAs, finding that increasing Al triggers a transition from FCC to FCC + BCC/B2 structures and shifts the morphology from columnar to equiaxed grains (Figure 10a). In Al0.75 and Al1.0 alloys, typical eutectic microstructures formed, consisting of lamellar/cellular FCC matrices and inter-dendritic B2 matrices with embedded BCC nanoprecipitates. Higher Al content also shifted the cracking mechanism from hot cracking to cold cracking, whereas the eutectic structure helped suppress crack initiation.
(2) Effect of Ni Content: Luo et al. [70] designed AlCrCuFeNix (2.0 ≤ x ≤ 3.0) alloys. Increased Ni content promoted the Columnar-to-Equiaxed Transition (CET) (Figure 10b), improving SLM formability. The AlCrCuFeNi3.0 alloy exhibited excellent nano-lamellar or cellular eutectic structures, resulting in a superior strength-ductility combination (UTS of 957 MPa, 14.3% elongation).

#### 3.2.2. Machine Learning in Composition Design

Beyond traditional semi-empirical rules, machine learning (ML) has recently emerged as a transformative tool for navigating the vast compositional space of HEAs. Modern ML workflows typically integrate high-throughput data mining with advanced algorithms such as Extreme Gradient Boosting (XGBoost), Back-propagation Neural Networks (BPNN), and Random Forests to predict phase stability and mechanical properties. A significant recent advancement by Shen et al. [71] demonstrated a machine learning-assisted design strategy for BCC refractory HEAs. By employing SHAP (Shapley Additive Explanations) feature importance analysis on a dataset of as-cast alloys, they identified that shear modulus mismatch and Molybdenum (Mo) content are the primary descriptors governing yield strength. This data-driven approach facilitated the development of a novel Ti-Mo-based HEA with a superior yield strength of 1169.3 MPa and 18.8% elongation, showcasing the power of ML in accelerating the discovery of alloys with optimized strength-ductility trade-offs. Such techniques are increasingly being adapted to SLM processes to optimize laser parameters and predict the formation of eutectic lamellae in Co-free systems.

Due to the vast compositional space of HEAs, machine learning (ML) is increasingly used to predict phase composition and properties efficiently [72].

(1). Phase Composition Prediction

**Figure 11:**
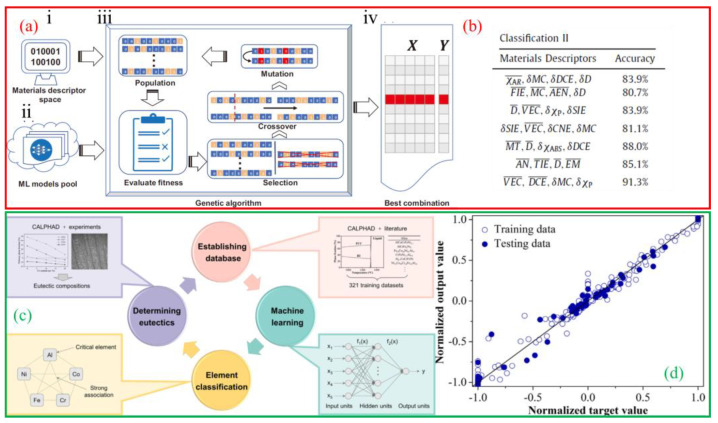
ML prediction of phase composition of HEAs: (**a**) model ((i) A materials descriptor space and (ii) a machine learning models pool are input into (iii) a GA iterative loop to search for the global optima by maximizing/minimizing a fitness function (e.g., the accuracy for classification or the root mean square error for regression) iteratively. Based on a stoping criterion on the performance of the fitness function, the GA outputs (iv) the most appropriate combination of materials descriptors and machine learning model), and (**b**) accuracy of phase prediction of as-cast HEAs [73]; phase prediction model (**c**) and prediction results (**d**) of Al-Co-Cr-Fe-Ni high-entropy system [74].

Within the framework of Materials Genome Engineering (MGE), Zhang et al. [73] achieved a 91.3% accuracy in predicting the phase composition of as-cast HEAs by selecting appropriate descriptors (Figure 11a,b). Wu et al. [74] established an ML-based design method for EHEAs, with experimental validation confirming excellent mechanical properties (Figure 11c,d).

(2). Performance Prediction

**Figure 12:**
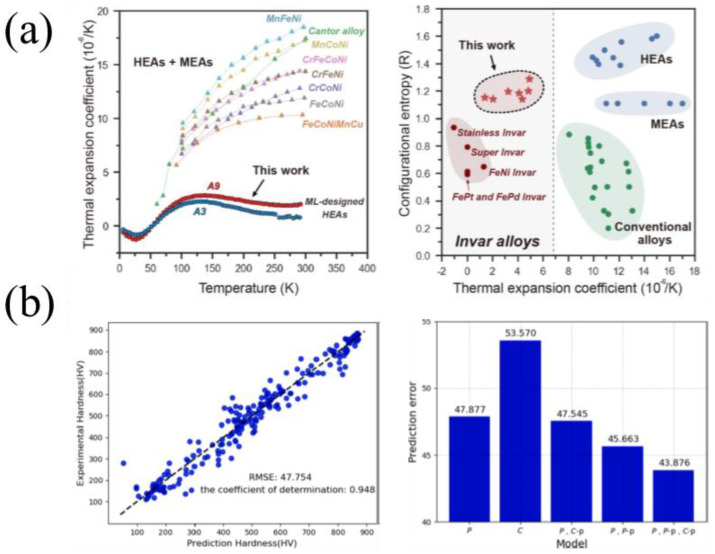
ML prediction of HEA properties: (**a**) low thermal expansion coefficient and high entropy Invar alloy [72]; (**b**) hardness of Al-Co-Cr-Cu-Fe-Ni HEAs [75].

Rao et al. [72] used an active learning strategy combining ML and Density Functional Theory (DFT) to design HEA Invar alloys with low thermal expansion coefficients (Figure 12a). Li et al. [75] optimized ML models using genetic algorithms to reduce error in hardness predictions for AlCoCrCuFeNi HEAs (Figure 12b). While thermal expansion and hardness predictions are well-reported, predicting tensile properties remains less explored despite its engineering importance.

In conclusion, combining experimental research with ML-driven predictions allows for rapid optimization of alloy compositions, facilitating the development of high-performance EHEAs for SLM applications like high-temperature tooling.

#### 3.2.3. Machine Learning Framework for Co-Free EHEAs

To navigate the vast compositional space of Cobalt-free EHEAs, machine learning (ML) has transitioned from a supportive tool to a core design methodology.

(1) Methodologies and Workflow

A typical ML workflow in this field involves: (1) Data Acquisition, integrating high-throughput CALPHAD calculations with experimental datasets from literature; (2) Feature Engineering, where physical descriptors are selected to represent the atomic environment; and (3) Model Training, employing algorithms such as Random Forest (RF) for high-dimensional data, or XGBoost for better handling of small, imbalanced datasets typical of new alloy systems.

(2) Input/Output Parameters and Descriptors

Critical to model accuracy is the selection of Input Parameters (Descriptors). Beyond simple atomic fractions, effective models now incorporate:

Electronic Descriptors: Valence electron concentration (VEC) and electronegativity difference (δ).

Geometric Descriptors: Atomic size difference (δ) and the γ parameter.

Thermodynamic Descriptors: Mixing enthalpy (δ_Hmix_) and configurational entropy (δ_Sconf_).

The Output Parameters typically focus on predicting the phase constitution (e.g., probability of forming a FCC + B2 eutectic structure) or specific mechanical properties like yield strength and microhardness [73].

(3) Practical Relevance: Case Studies

Compositional Screening: Recent work by Shen et al. [71] utilized SHAP (Shapley Additive Explanations) analysis to identify that “shear modulus mismatch” is a dominant descriptor for strength in BCC-structured HEAs, leading to the rapid discovery of alloys that broke the strength-ductility trade-off.

SLM Process Optimization: ML is also applied to predict the Printability Window. By using laser power and scanning speed as inputs, Gaussian Process Regression (GPR) models can map the density of Co-free AlCrFeNi alloys, reducing experimental iterations by over 70%.

(4) Limitations and Challenges

Despite its potential, ML in HEA research faces several bottlenecks:

Data Scarcity: Experimental data for Co-free systems is still sparse compared to Co-containing ones, leading to potential model bias.

Extrapolation Risks: ML models often struggle to predict properties of alloys that lie far outside the training distribution (e.g., transitioning from Al-Cr-Fe-Ni to new refractory systems).

Physical Interpretability: “Black-box” models may provide accurate predictions without revealing the underlying metallurgical mechanisms, necessitating the use of explainable AI (XAI) tools [74].

### 3.3. Research Status of Microstructural Evolution

The composition design of high-entropy alloys significantly influences the microstructures and properties of SLM-fabricated EHEAs. The microstructures of these alloys primarily manifest as lamellar, cellular, or a coexistence of both. Among these, lamellar structures have been the most extensively researched, as summarized in Table 4.

#### 3.3.1. EHEAs with Lamellar Eutectic Structures

**Figure 13:**
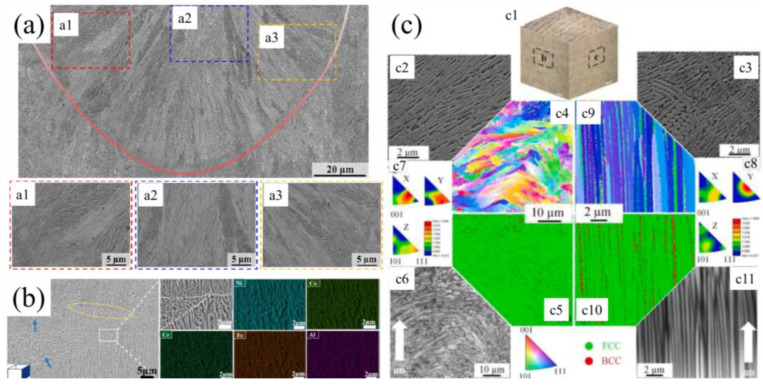
Microstructure of SLM-processed AlCoCrFeNi2.1 microstructure: (**a**) (a1–a3, Magnified images showing regions with different shape orientations of nanolamellar eutectic colonies inside the melt pool.) [25]; (**b**) (the fusion line was marked by the blue arrow) [77]; (**c**) ((c1) The OM images of SLM printed sample; (c2,c3) the SEM images of side faces; EBSD results of AlCoCrFeNi2.1 EHEA using VED of 92.6 J/mm^3^ of the SLM printed sample cross-section along the BD under different magnification: (c4) orientation maps; (c5) phase maps; (c6) FSD and (c7,c8) IPF plots under magnification of 1000×; (c9) orientation maps; (c10) phase maps and (c11) FSD under magnification of 5000×) [76].

Numerous studies indicate that SLM-fabricated AlCoCrFeNi2.1 EHEAs frequently exhibit lamellar eutectic microstructures [25,76,77] (see Figure 13a–c). Niu et al. [78] utilized SLM to prepare AlCoCrFeNi alloys where the eutectic structure consisted of BCC and B2 phases; the microstructure featured BCC columnar grains oriented perpendicular to the melt pool boundary, with an average grain size of approximately 1.5 μm, and the B2 phase distributed between these columnar grains. Yang et al. [8] successfully fabricated crack-free Ni30Co30Cr10Fe10Al18W1Mo1 alloys via SLM, characterized by ultrafine eutectic lamellar spacing of 150–200 nm and grain sizes of 2–6 μm. Sun et al. [33] found that the microstructure of SLM-fabricated Al0.5CoCrFeNi consisted solely of FCC and BCC phases, with no complex intermetallic compounds detected. Furthermore, Ren et al. [4] observed that AlCoCuFeNi alloys mainly comprised a columnar BCC matrix and a Cu-rich FCC phase, displaying a distinct solidification texture.

#### 3.3.2. EHEAs with Cellular Eutectic Structures

Vogiatzief et al. [14] employed SLM to produce Al0.9Cr0.9Fe2.1Ni2.1 alloys, resulting in a metastable FCC structure. Upon heat treatment, the BCC/B2 phases became uniformly dispersed within the FCC matrix. Jung et al. [79] discovered that AlCoCrFeMnNi alloys formed a nanoscale modulated structure during the SLM process, composed of Al/Ni-rich B2 phases and Cr/Fe-rich BCC phases.

#### 3.3.3. EHEAs with Coexisting Lamellar and Cellular Structures

**Figure 14:**
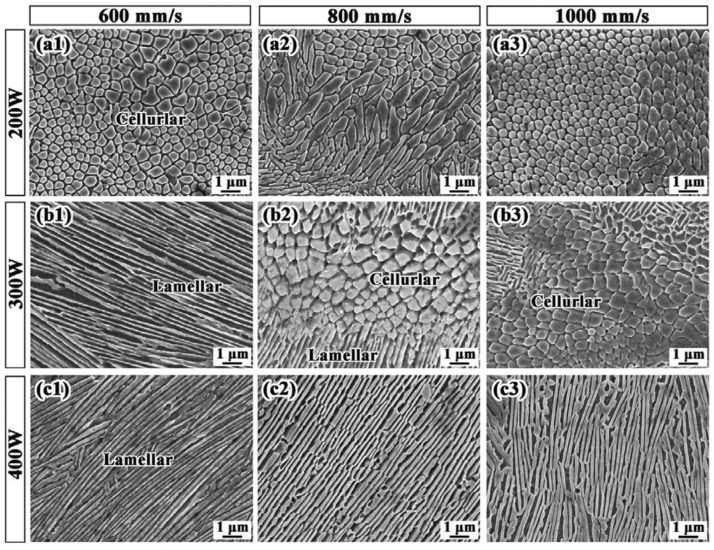
Microstructure evolution of AlCoCrFeNi2.1 EHEA as functions of laser power of (**a**) 200 W, (**b**) 300 W, (**c**) 400 W, and scanning speed of (1) 600 mm/s, (2) 800 mm/s and (3) 1000 mm/s [26].

Guo et al. [26] investigated SLM-fabricated AlCoCrFeNi2.1 and found that as the laser energy density decreased, the eutectic microstructure gradually evolved from a lamellar to a cellular morphology (Figure 14). Luo et al. [70] designed and manufactured FCC + BCC dual-phase EHEAs, observing the formation of modulated nanoscale lamellar or cellular eutectic structures. Su et al. [7] noted that increasing the Al content triggered a transition from a single FCC phase to a dual-phase FCC + BCC/B2 structure, accompanied by an evolution from columnar to equiaxed grains.

In summary, SLM-fabricated EHEAs typically exhibit lamellar eutectic structures, though some alloys display cellular or mixed morphologies. For Cu-containing HEAs, a higher FCC phase content generally correlates with improved crack resistance. Furthermore, simulating the temperature field of the SLM melt pool in conjunction with CALPHAD (Calculation of Phase Diagrams) can better elucidate the evolution of microstructural morphology and the specific influence of processing parameters.

### 3.4. Non-Equilibrium Solidification Physics in SLM of Co-Free EHEAs

The hallmark of SLM is its extreme thermal cycle, characterized by cooling rates reaching 10^5^–10^8^ K/s. In Cobalt-free eutectic systems (e.g., Al-Cr-Fe-Ni), these conditions trigger a departure from equilibrium solidification, fundamentally altering the competitive growth between phases.

#### 3.4.1. Suppression of Primary Phase Growth

Under equilibrium conditions, even slight deviations from the precise eutectic composition often lead to the formation of coarse primary dendrites (e.g., BCC-B2 or FCC phases). However, the ultra-fast cooling in SLM extends the “Eutectic Coupled Zone.” The high undercooling (ΔT) achieved at the liquid-solid interface increases the growth velocity of the eutectic interface beyond the competitive threshold of primary dendrites. This effectively suppresses the precipitation of pro-eutectic phases, allowing for a fully eutectic microstructure even in slightly off-eutectic Co-free compositions.

#### 3.4.2. Formation of Sub-Micron to Nanoscale Eutectic Lamellae

The relationship between the eutectic interlamellar spacing (λ) and the growth velocity (V) is governed by the Jackson-Hunt theory, typically expressed as λ^2^ V = constant. In SLM-processed Co-free EHEAs, the extreme V forced by rapid heat extraction leads to:

Significant Structural Refinement: The lamellar thickness is reduced from tens of microns (in traditional casting) to 100–300 nm.

Solute Trapping Effect: The absence of Cobalt modifies the diffusion coefficients of remaining elements like Al and Cr. At these high velocities, “solute trapping” occurs, where atoms are “frozen” into the crystal lattice before they can partition between the FCC and B2 phases. This leads to the formation of supersaturated solid solutions and highly refined hierarchical structures that are unattainable through conventional processing.

#### 3.4.3. Impact of the Absence of Cobalt on Solidification Morphology

Without the moderating effect of Cobalt on the melting range and thermal conductivity, Co-free melts exhibit a unique Melt Pool Dynamics. The increased temperature gradient (G) and growth rate (R) ratio (G/R) at the melt pool boundary promote a transition from cellular to fine equiaxed eutectic grains. This microstructural heterogeneity, characterized by nanolamellar clusters within ultrafine grains, is the primary driver for the superior strength-ductility synergy observed in these Co-free systems.

### 3.5. Research Status of Performance Tuning Technologies for SLM-Fabricated EHEAs

Eutectic high-entropy alloys (EHEAs) fabricated via SLM are often accompanied by high residual stresses, necessitating post-heat treatment to release these stresses and optimize the strength-ductility balance [81]. Table 5 summarizes typical heat treatment methods for SLM-fabricated EHEAs and their effects on microstructures and mechanical properties. The critical parameters in post-heat treatment are temperature and cooling rate; their respective influences on microstructure and properties are summarized below.

#### 3.5.1. Influence of Post-Heat Treatment Temperature

**Figure 15:**
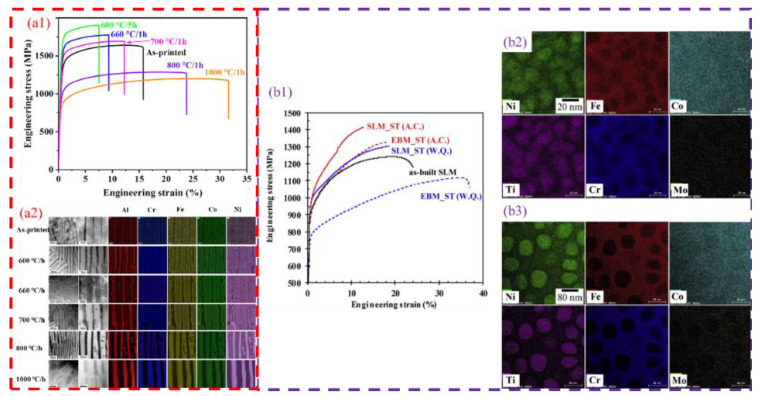
Heat treatment of as-SLMed EHEAs: (**a1**) tensile properties and (**a2**) element distribution of as-SLMed and annealed AlCoCrFeNi2.1 [25]; (**b1**) tensile stress–strain curves, and element distribution of Co1.5CrFeNi1.5Ti0.5Mo0.1 obtained by (**b2**) water cooling and (**b3**) air cooling [43].

Research indicates that low-temperature annealing (600–700 °C) can promote the formation of precipitates and alter the phase structure. In a study of AlCoCrFeNi2.1 alloys, Ren et al. [25] found that precipitates formed at 700 °C significantly increased the alloy’s strength, though at the expense of ductility. As the temperature rose to 800 °C, these precipitates vanished, leading to a decrease in strength and an improvement in ductility (Figure 15(a1,a2)). Other researchers [8,14,80] have similarly demonstrated that annealing above 900 °C significantly improves elongation while reducing strength. These findings emphasize that heat treatments above 800 °C typically help reduce strength in favor of enhanced ductility, whereas treatments below 800 °C favor the formation of ordered phase structures to enhance strength.

#### 3.5.2. Influence of Cooling Methods

The cooling method plays a vital role in regulating the properties of SLM-fabricated EHEAs. As shown in Table 5, while air cooling is the most prevalent method, water quenching can also significantly improve performance. Research by Fujieda et al. [42] demonstrated that water quenching could enhance the tensile properties and pitting corrosion resistance of CoCrFeNiTi alloys. This suggests that water cooling, as a rapid cooling strategy, holds significant potential for tuning the performance of specific high-entropy alloys (Figure 15(b1–b3)).

In conclusion, the heat treatment temperature for SLM-fabricated EHEAs is generally selected within the range of 600–1200 °C. Processing above 800 °C typically results in increased ductility and decreased strength, while air cooling remains the most common cooling method. Research on water cooling is relatively scarce and warrants further exploration.

## 4. Summary

This review has systematically summarized the recent progress in the design, fabrication, and performance control of Co-free EHEAs via SLM. Key conclusions include:

Compositional Advantages: The transition to Co-free systems, primarily through the optimization of Al, Ni, and Fe ratios, offers a sustainable and cost-effective pathway for high-temperature structural materials.

Microstructural Control: SLM’s ultra-high cooling rates enable the formation of unique nano-lamellar and cellular eutectic structures that are unattainable through conventional casting. These structures, particularly the FCC + BCC/B2 architectures, provide a superior strength-ductility balance, with yield strengths exceeding 1000 MPa and elongations reaching 15–20%.

Post-processing Synergy: Thermal treatments above 800 °C facilitate the dissolution of brittle phases and the reduction in dislocation density, significantly recovering ductility (up to a 15-fold increase) while only moderately sacrificing strength. Rapid cooling strategies like water quenching show immense promise in enhancing both mechanical toughness and pitting corrosion resistance.

## 5. Future Perspectives

Despite significant advances, several challenges remain for the industrial deployment of SLM-fabricated Co-free EHEAs:

Standardization of Co-free Systems: A unified definition and a larger database for “high-performance Co-free” compositions are needed. Future research should leverage Machine Learning not just for phase prediction, but for the multi-objective optimization of tensile properties and long-term thermal stability.

Mechanistic Understanding of Cooling Rates: The impact of water cooling vs. air cooling on the formation of Cr-depleted zones and subsequent corrosion behavior requires more granular TEM and atom probe tomography (APT) studies.

Process-Structure-Property Maps: Future work must bridge the gap between SLM parameters (e.g., VED) and the resulting hierarchical microstructure to enable “first-time-right” printing of complex, crack-free eutectic components.

## 6. Current Challenges and Limitations

While SLM-processed Co-free EHEAs exhibit exceptional laboratory-scale properties, several critical challenges must be addressed before their widespread industrial adoption.

### 6.1. Defect Sensitivity and Cracking Mechanisms

The inherent high cooling rates and steep thermal gradients (10^6^–10^7^ K/m) in SLM induce significant residual stresses. In Co-free systems, the absence of Cobalt can alter the liquid-solid surface tension and the solidification temperature range. This often leads to increased susceptibility to hot cracking (at the final stages of solidification) and solid-state cracking (due to the brittle nature of the B2 phase). Controlling the “Printability Window” requires a delicate balance between laser power and scanning speed to avoid porosity from both “lack-of-fusion” and “keyhole” regimes.

### 6.2. Compositional Homogeneity and Elemental Evaporation

The high energy density of the laser beam causes selective evaporation of volatile elements with high vapor pressures, such as Aluminum (Al) and Manganese (Mn). In Eutectic HEAs, even a minor loss of Al can shift the composition away from the eutectic point, leading to the formation of primary dendrites instead of the desired fine lamellar structure. This compositional drift complicates the predictability of mechanical properties across large-scale components.

### 6.3. Scalability and Geometric Constraints

Current research is largely limited to small-scale coupons (10 × 10 × 10 mm^3^). Scaling up to large structural components introduces cumulative thermal stress, which increases the likelihood of part distortion or delamination from the build plate. Furthermore, the recyclability of Co-free HEA powders and the consistency of properties in complex thin-walled geometries remain insufficiently explored.

### 6.4. Long-Term Stability in Extreme Environments

Although these alloys are designed for extreme environments, there is a lack of long-term data regarding their creep resistance and oxidation kinetics under cyclic thermal loading. The high density of phase boundaries in nanolamellar EHEAs provides a high driving force for grain coarsening at elevated temperatures, potentially compromising their structural integrity over extended service lives.

## Figures and Tables

**Table 2:** Phase formation and tensile properties at room temperature of SLM-processed HEAs that have been reported (VED: volume energy density).

HEA Chemical Formula	VED (J/mm^3^)	Phase	Elongation (%)	Ultimate Tensile Strength (MPa)	Yield Strength (MPa)	References
CoCrFeMnNi	60	FCC	34	609	510	[29]
CoCrFeMnNi	89	FCC	23	650	550	[30]
FeCoCrNi	168.9	FCC	19	700	580	[31]
FeCoCrNi	95.24	FCC	45.3	714	585	[12]
Al0.5FeCoCrNi	69.4	FCC	50	721	579	[33]
CoCr2.5FeNi2TiW0.5		BCC	9.9	893	581	[33]
Al0.5CoCrFeNi	150	FCC	17	878	609	[34]
FeCoCrNi	100	FCC	32	745	600	[18]
Al0.3CoCrFeNi	129	FCC	29	896	730	[35]
FeCoNiCr0.5	160.71	FCC	28	642	547	[19]
CrCoNi	109.1	FCC	35.9	907.7	651	[36]
FeCrNi	90.9	FCC	31	1007	745	[24]
Ni10Cr6WFe9Ti	57.36	FCC	26.5	961.55	739.77	[37]
Fe50Mn30Co10Cr10	105.82	FCC	32.5	744.9	487.6	[38]
Al0.3Ti0.2Co0.7CrFeNi1.7	75	FCC	15	787	600	[39]
Al0.3Ti0.2Co0.7CrFeNi1.7	62.5	FCC	15	773	600	[39]
Fe60(CoCrMnNi)40	158.73	FCC	58	573	417	[40]
(CoCrNi)94Al3Ti3	97.22	FCC	30.7	1027	772	[41]
Fe30Mn50Co10Cr10	105.82	FCC	22.3	687.7	580.65	[38]
Fe50Mn30Co10Cr10	91.67	FCC	18.7	630	266	[20]
CoCrFeMnNi + 3wt%Ti	88.9	FCC	22	760.8	585.8	[42]
Co1.5CrFeNi1.5Ti0.5Mo0.1	61.5	FCC	25.8	1178	773	[43]
Al0.2Co1.5CrFeNi1.5Ti0.3	74.3	FCC + precipitates	10	500	900	[44]
CoCrFeNiTiMo	83.3	FCC + precipitates	20	1150	860	[45]
(CoCrNi)95Mo5	138.8	FCC +precipitates	35.9	970	790	[36]
(CoCrNi)95Mo5	104.2	FCC + precipitates	33.1	990	800	[46]
(CoCrNi)95Mo5	83.3	FCC + precipitates	30.1	980	800	[46]
(CoCrNi)95Mo5	69.4	FCC + precipitates	28.3	960	790	[46]
(FeCoNi)85.84Al7.07Ti7.09	88.89	FCC + precipitates	11.6	1625	1214	[47]
CoCrFeNiTi0.3		FCC + precipitates	3.7	796		[48]
Ni40Co18Cr18Fe14Al5Ti5	117.19	FCC + precipitates	26.9	1073.5	812.5	[49]
Ni40Co18Cr18Fe14Al5Ti5	93.75	FCC + precipitates	28.6	1062.5	790.4	[49]
Ni40Co18Cr18Fe14Al5Ti5	62.5	FCC + precipitates	28.8	1072.6	807.5	[49]
AlCoCrFeNi2.1	92.6	FCC + BCC	13	1386	1063	[25]
AlCoCrFeNi2.1	121.21	FCC + BCC	21.8	1271	966	[26]
AlCoCrFeNi2.1	126.98	FCC + BCC	7.5	1275	975	[26]
AlCoCrFeNi2.1	141.41	FCC + BCC	23.5	1271	966	[26]
AlCoCrFeNi2.1	166.67	FCC + BCC	6	1490	1200	[26]
AlCoCrFeNi2.1	176.77	FCC + BCC	4	1420	1210	[26]
AlCoCrFeNi2.1	64.8	FCC + BCC	12.3	1322.8	982.1	[27]
CrFeCoNiAl0.4Ti0.14	156.25	FCC + BCC	3	1020	850	[50]
Al0.7CoCrFeNi2.4	71.4	FCC + BCC	24.8	1226.3	943.2	[50]
Al0.7CoCrFeNi2.4	89.3	FCC + BCC	27.4	1155.6	917.5	[50]
Al0.7CoCrFeNi2.4	100	FCC + BCC	23.7	1172.6	915.7	[50]
Al0.7CoCrFeNi2.4	119	FCC + BCC	26.6	1142.5	904.4	[50]
Al0.7CoCrFeNi2.4	125	FCC + BCC	22.8	1188.3	937.5	[50]
Al0.7CoCrFeNi2.4	166.7	FCC + BCC	17.9	1102.2	957.5	[50]
(FeCrNi)94Ti3Al3	75.76	FCC + BCC	13	1421	948	[51]
Al18Co30Cr10Fe10Ni32	92.59	FCC + BCC	21.3	1368.6	1064.3	[28]
AlCoCrFeNi2.5	107.13	FCC + BCC	18.3	1088	926	[52]
Ni30Co30Cr10Fe10Al18W1Mo1		FCC + BCC	20	1400	1000	[8]

**Table 3:** Preparation methods of HEA (some are EHEAs) powder for SLM other than gas atomization.

HEA Chemical Formula	Powder Preparation	Reference
CoCrFeMnNi	CoCrFeNi + Mn	[67]
Al0.9Cr0.9Fe2.1Ni2.1	AlCrFe2Ni2 + Fe + Ni	[14]
HEA-TiAl	CoCrFeMnNi + Ti48Al2Cr2Nb	[68]
(Fe50Mn30Co10Cr10)100-xSix (x = 0, 1, 3, 5)	Fe50Mn30Co10Cr10 + Si	[20]
CrMoTi	Cr + Mo + Ti	[11]
Al0.5CoCrFeNi	Al + Co + Cr + Fe + Ni	[34]

**Table 4:** SLM formability and microstructure characteristics of EHEAs.

HEA Chemical Formula	SLM Printability	Microstructural Characteristics	Reference
AlCoCrFeNi2.1	RD 99.73%	Lamellar (FCC + B2) or Cellular (FCC encapsulated by B2)	[26]
AlCoCrFeNi2.1	RD 99.10%	Lamellar (FCC + B2)	[76]
AlCoCrFeNi2.1	RD 99.3%	Lamellar (FCC + B2)	[27]
AlCoCrFeNi2.1	/	Lamellar (FCC + B2)	[25]
AlCoCrFeNi2.1	/	Lamellar (FCC + B2)	[77]
AlCoCrFeNi	RD 98.4%	Lamellar (BCC + B2)	[78]
Ni30Co30Cr10Fe 10Al18W1Mo1	Minimum porosity <0.05%	Lamellar (FCC + B2)	[8]
Al0.5CoCrFeNi	RD 99.92%	Lamellar (FCC + B2)	[38]
Al0.9Cr0.9 Fe2.1Ni2.1	Minimum porosity: 0.061%	Cellular (BCC encapsulated by FCC)	[14]
AlCoCrFeMnNi	Cracking	Cellular (B2 encapsulated by BCC)	[79]
AlCoCuFeNi	Cracking	BCC, negligible FCC	[4]
AlCoCuFeNi	/	B2/BCC	[80]
Al0.75CrCuFeNi2	No cracking	Lamellar or cellular (BCC/B2 encapsulated by FCC)	[7]
AlCrCuFeNi2	Cold cracking	Lamellar or cellular (BCC/B2 encapsulated by FCC)	[7]
AlCrCuFeNi3.0	RD 99.7%	Lamellar or cellular (BCC/B2 encapsulated by FCC)	[21]

**Table 5:** Post-heat treatment and its effects on microstructure and properties of as-SLMed EHEAs.

HEA Chemical Formula	Heat Treatment Method	Microstructural Evolution	Mechanical Property Evolution	Reference
AlCoCrFeNi2.1	600 °C/5 h, 660 °C/1 h, 700 °C/1 h, Water quenching	BCC: precipitation of needle-like precipitates rich in Co, Cr, and Fe, as well as Cr-rich spherical nano-precipitates, 700 °C/1 h annealed sample: the formation of Ni-Al-rich blocky nano-precipitates within the FCC matrix	Strength-ductility trade-off, where the yield strength increases significantly at the expense of ductility	[25]
AlCoCrFeNi2.1	800 °C/1 h, Water quenching	The precipitates in the BCC phase vanished, while the blocky Ni-Al-rich nanoprecipitates in the FCC phase underwent coarsening	Strength-ductility trade-off, where ductility increases significantly at the expense of the yield strength	[25]
AlCoCrFeNi2.1	1000 °C/1 h, Water quenching	The amount of precipitation decreased, and the elemental distribution within each phase became more uniform	The dislocation density decreased, leading to improved ductility and a reduction in strength	[25]
Ni30Co30Cr10Fe10Al18W1Mo1	900 °C/2 h, Air cooling	No significant change	The elongation increased from 1% to over 15%	[8]
Al0.9Cr0.9Fe2.1Ni2.1	950 °C/6 h, Air cooling	Formation of BCC/B2 precipitates	The elongation increased to 20% (from an initial 15%)	[14]
AlCoCuFeNi	900/1000 °C/10 h, Air cooling	Precipitation of the FCC phase from the BCC (B2) matrix	The microhardness and compressive yield strength decreased, while the ductility was significantly improved	[80]
Co1.5CrFeNi1.5Ti0.5Mo0.1	1120 °C/3 h, Air cooling or water quenching	Nanoscale precipitates (tens of nanometers in size)	The tensile and pitting corrosion resistance of the water-quenched samples were significantly improved	[43]

## Data Availability

The data that support the findings of this study are available from the corresponding author upon reasonable request.
